# Using Young Tongue-Holding Forceps for Third Molar Autotransplantation as a New Method for Tooth Handling: A Case Report with a 1-Year Follow-Up -

**DOI:** 10.31661/gmj.vi.3932

**Published:** 2025-09-22

**Authors:** Hadi Shakerin, Mohammad Reza Sadeghi, Ahmad Toumaj, Zahra Jafary Nodoushan

**Affiliations:** ^1^ Department of Endodontics, School of Dentistry, Baqiyatallah University of Medical Sciences, Tehran, Iran; ^2^ Department of Periodontics, School of Dentistry, Baqiyatallah University of Medical Sciences, Tehran, Iran; ^3^ Department of Prosthodontics, Dental School, Islamic Azad University of Medical Sciences, Tehran, Iran

**Keywords:** Autologous Transplantation, Tooth Transplantation, Third Molar, Autotransplantation, Young Tongue Holding Forceps

## Abstract

For the first time in autotransplantation literature, all extraoral procedures on the donor tooth were carried out while holding it by a Young Tongue Holding Forceps, potentially reducing damage to the PDL and enhancing the handling of the tooth. In this case report, we present the autotransplantation of a mandibular third molar into the socket of the adjacent second molar in an 18-year-old patient. The clinical and radiographic evaluation revealed severe pain and a periapical lesion from the previous root canal treatment. The surgical and endodontic steps were carried out with a focus on maintaining the viability of periodontal ligament cells, which would improve the overall prognosis of the transplantation. The donor tooth was secured in the ideal position using sutures for two weeks. The transplanted tooth was followed up for 12 months postoperatively, showing optimal healing and maturation of periodontal and periapical tissues. It seems that Young Tongue Forceps could be used in other dental practices that involve ex vivo or extraoral procedures on a tooth. Furthermore, the forceps could be modified and manufactured to accommodate every type of tooth.

## Introduction

Replacement of the permanent teeth with an unfavorable prognosis remains one of the main foci of ongoing research and development in the dental field. Dental caries, trauma, agenesis, and other factors and scenarios may necessitate replacement of teeth, for which many options exist. Among the most frequently chosen clinical options to replace teeth are removable and fixed prostheses, including bridges and implants [1]. Besides the aforementioned conventional options, there are less obvious options that are best suited for specific clinical scenarios but might yield similar or even superior results in some aspects [2].

One of the most prominent of these options is Tooth Autotransplantation (TAT). The main advantages of TAT stem from the fact that TAT is a biologic replacement for lost teeth, which is in direct contrast to conventional methods such as bridges and implants that utilize mechanical means to restore teeth. This biologic aspect is a result of preservation of the periodontal ligament (PDL) and pulp viability. Preservation of PDL allows for continued eruption and future orthodontic movement of the replaced teeth, preserves the proprioception and the sensory apparatus of the periodontal ligament, and helps maintain the gingiva and alveolar bone volume. If the transplanted teeth have incomplete root formation, preservation of pulp vitality allows for the completion of root formation and closure of apical foramina [3][4].

Advantages mentioned above, in addition to relatively lower cost[5][6] and shorter treatment and chairside time [6], have made TAT an optimal choice under the right clinical conditions. One of the main indications of TAT is incomplete skeletal development in the patient, which is a contraindication for bridges and dental implants [6]. The transplanted tooth has a high chance to continue its root development and will continue to migrate and erupt similarly to the rest of the dentition [7][8].

In this case report, we will present and discuss a case of TAT in a young patient in which a permanent lower second molar was replaced by the adjacent third molar.

## Case Report

**Figure-1:**
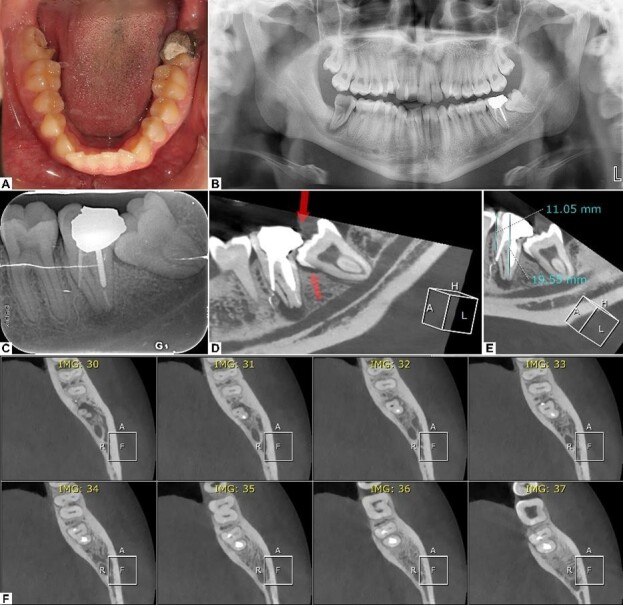
Figure 1. (A) Intraoral photograph of the patient’s mandibular dentition. (B) Panoramic radiograph. (C) Periapical radiograph, demonstrating previous suboptimal root canal therapy. (D) CBCT slice depicting extensive periapical lesion. (E) Recipient site measurements. (F) Axial CBCT reconstruction showing lateral root perforation and missed mesiolingual canal in the second molar

**Figure-2:**
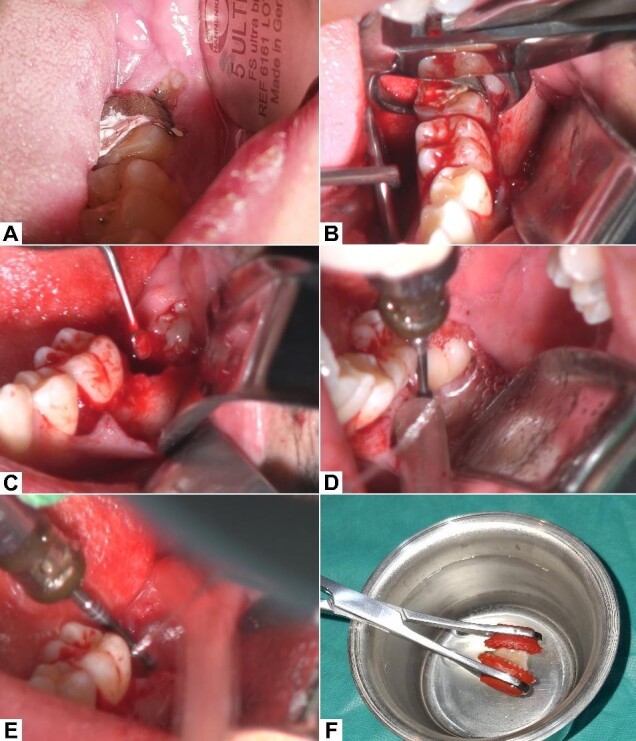
Figure 2. (A) Initial intraoral presentation; the crown of the third molar is partially visible through the gingiva. (B) Extraction of the second molar after flap reflection. (C) Extraction site debridement and curettage to remove all granulation tissue. (D) Buccal troughing and bone removal prior to the extraction of the third molar. (E) Recipient site preparation according to the donor tooth dimensions. (F) Extracted donor tooth being held extraorally using Young’s Tongue Forceps.

**Figure-3:**
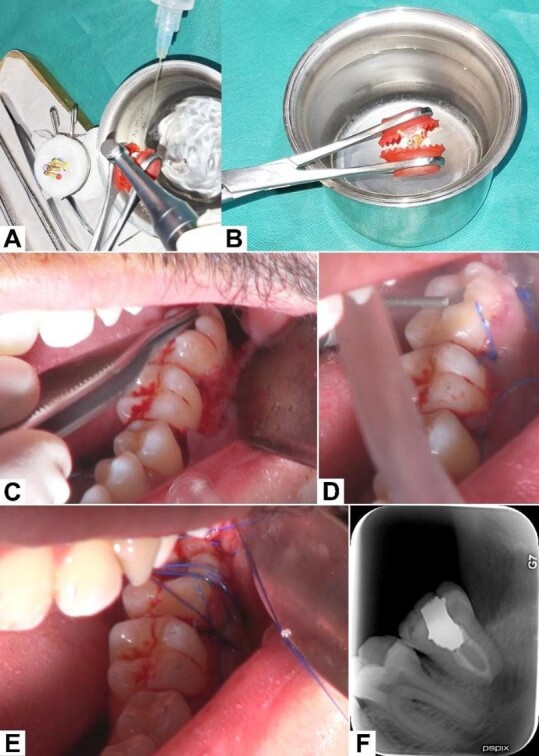
Figure 3. (A) Cleaning and shaping of the root canal system with constant irrigation of the root portion to help maintain PDL vitality. (B) Obturation via lateral condensation technique. (C) Donor tooth positioned in the recipient socket. (D) Flap closure and occlusal adjustment. (E) Horizontal mattress sutures passed over the crown of the donor tooth to help stabilize it in position. (F) Post-op periapical radiograp.

**Figure-4:**
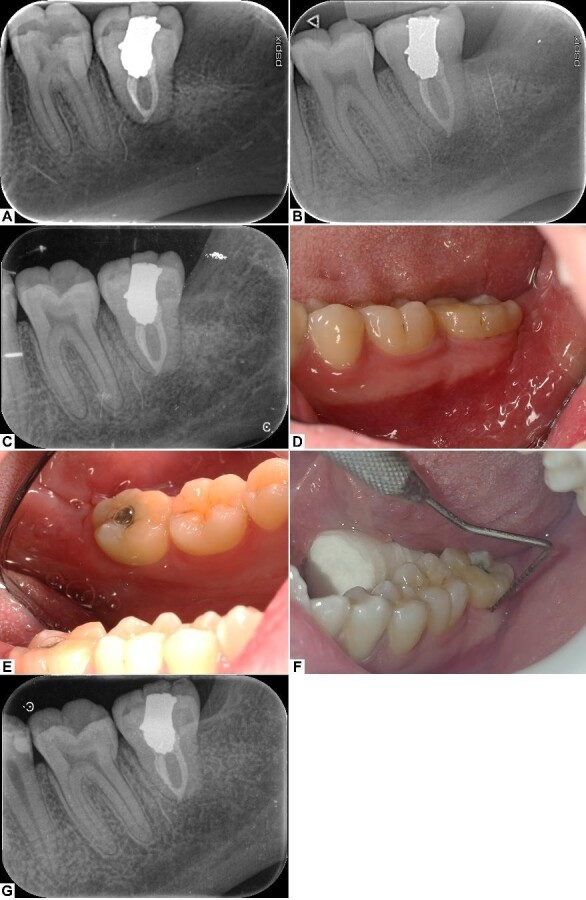
Figure 4. Periapical radiographs at 2 weeks (A), 3 months (B), 6 months (C) post-op; progressive healing and maturation of the periodontal ligament and lamina dura are seen. Intraoral photographs at 6 months post-op from the buccal (D) and lingual (E) sides, showing healthy soft tissue with no visible inflammation of the gingiva. (F) Normal probing depths around the transplanted tooth. (G) Final periapical radiograph at 12 months post-op.

### Case Presentation

An 18-year-old male patient was referred with the chief complaint of severe pain in the left posterior mandible, which had started two days prior to the visit. The referring dentist ordered a Cone-Beam Computed Tomography (CBCT) to attempt endodontic retreatment. Endodontic retreatment was aborted before completion of access cavity preparation, and the patient was subsequently referred to the current center.

Nothing of significance was found in the patient’s medical history. In the dental examination, the lower left second molar had a history of root canal treatment (RCT) 2.5 years ago, an amalgam filling with signs of recurrent caries underneath, and an incomplete access cavity prepared through the amalgam restoration filled with a temporary filling material (which was the result of the previous attempt by the referring dentist to perform non-surgical endodontic retreatment).

In the examination of the patient’s radiographs, the panoramic and periapical radiographs revealed a suboptimal RCT for the suspected tooth with an apparent lateral perforation of the mesial root. The CBCT radiograph not only confirmed the previous findings but also showed the extent of the periapical lesion and the lateral perforation and that the mesiolingual canal was completely missed in the previous RCT procedure (Figure-1).

A cold test for the assessment of tooth vitality was carried out on the suspected tooth and adjacent first molar. While the first molar responded normally to the cold test, the second molar did not respond to the cold test. Percussion and palpation tests for the first molar both yielded negative results, while the second molar had a strong positive response to the percussion test (++) and was tender to the vestibular palpation test (+).

Based on clinical findings and tests carried out, a pulpal diagnosis of "previous endodontic treatment with an infected root canal system" and a periapical diagnosis of "secondary acute apical periodontitis" were established (both according to the Abbott classifications [9][10]). The treatment options included retreatment, extraction and subsequent placement of a dental implant, and autotransplantation. When presented with the aforementioned options, the patient complained about the numerous previous procedures performed on this specific tooth and stated their frustration and unwillingness to attempt to retain the second molar. Based on the clinical status, the prognosis of retreatment, the young age of the patient and their incomplete skeletal growth, economic factors, poor periodontal prognosis, and the presence of a suitable adjacent third molar, the clinicians and the patient agreed on the choice of autotransplantation. Written informed consent was obtained from the patient regarding the treatment, and the surgery appointment was scheduled. Ibuprofen 400 mg three times per day and Acetaminophen 325 mg three times per day were prescribed for the patient to reduce the pain and inflammation. Based on the ESE position statement(2021) for tooth autotransplantation, in medically competent patients, there was no indication for antibiotic prophilaxis [11].

### Surgical Procedure

Anesthesia was achieved by inferior alveolar and long buccal nerve blocks using 2% lidocaine + 1:80000 epinephrine. A sulcular flap was made, and the hopeless second molar was extracted using mandibular cowhorn forceps. The extraction socket was thoroughly curetted to remove all residual granulation and inflamed tissue. Following curettage, the recipient socket was prepared using a round bur and copious saline irrigation as coolant to create space for the donor tooth. Following recipient socket preparation, a buccal bone trough was prepared around the third molar to ease its surgical extraction with caution not to harm the cementum. Following the extraction of the third molar, extraoral RCT was carried out (Figure-2).

Following the completion of the RCT, the third molar was replanted in the recipient socket and properly positioned. Flap margins were sutured by simple loop sutures using 5-0 nylon (Supalon; SUPA Medical, Tehran, Iran). Occlusal reduction and adjustment were done, and two horizontal mattress sutures were passed on top of the replanted tooth to act as a soft splint, which would help secure the tooth in place (Figure-3). No other form of splinting was used.

### Root Canal Treatment

The extracted third molar was held by a Young Tongue Holding Forceps (S-M Instruments, Pakistan) and continuously irrigated by sterile Hank’s Balanced Salts Solution (HBSS; Biowest, Nuaille, France) to help reduce damage to the periodontal ligament (PDL) and preserve its cellular viability. An access cavity was prepared, and straight-line access was established. Root canal preparation was done using NiTi rotary files (TG6; Shenzhen perfect Medical Instruments, Guangdong, China) and concomitant irrigation of the root canal system with 2.5% sodium hypochlorite and sterile saline. Root canal obturation was done via the single cone technique using gutta percha points 4% (Diadent,South Korea) and a cold ceramic sealer (CC Sealer; SAMIN, Yazd, Iran). The access cavity was filled with amalgam. The entirety of the RCT procedure took approximately 17 minutes (Figure-3).

### Post-op and follow-up

Post-operatively, the patient was instructed to maintain regular oral hygiene measures but to brush the surgical site very lightly. They were also instructed to eat a softer diet for the first few days, avoid hard or sticky foods, and avoid chewing with the left side for 2 weeks. They were also instructed to continue taking the prescribed analgesics whenever needed, and continue rinsing their mouth with chlorhexidine until suture removal [10]. Amoxicillin 500 mg TID was prescribed to prevent root resorption and increase the success rate [11][12].

The first follow-up appointment was scheduled two weeks post-surgery to assess healing and remove the sutures. Next follow-ups were scheduled for 3-, 6-, and 12-months post-surgery. A periapical radiograph was taken at each appointment to assess peri-radicular healing. The periodontal examination was performed by a UNC15 periodontal probe (Hu-Friedy, Illinois, US) using a light probing force of 0.25N. Pocket depth and bleeding on probing were assessed around the tooth.

### Outcome

The patient returned for suture removal on the 12th day after the surgery. Healing was uneventful, and the patient was asymptomatic. The periapical radiograph revealed early signs of periradicular healing, especially in the apical region (Figure-4.A).

At the 3-months post-op follow-up visit, the patient was asymptomatic, had no complaints, and the tooth was functioning normally. The periapical radiograph revealed noticeable healing and maturation of periradicular tissues, with a distinct PDL space and early formation of lamina dura (Figure-4.B).

The next follow-up visits were held 6 and 12 months after the surgery. Clinical examination yielded nothing of significance; the gingiva was healthy and free of inflammation, probing depth was within normal limits (Figure-4D and 4E and 4F), the tooth was not tender to percussion or palpation, and it had normal mobility according to the Miller classification [13]. On radiographic examination, a fully formed lamina dura and a distinctive PDL space could be observed. No pathologic periapical radiolucency was observed, and the peri-radicular and crestal bones were normal (Figure-4C). The 1-year post-op periapical radiograph showed proper healing and no signs of negative outcomes such as external root resorption (Figure-4-G).

### Ethical Approval

Written informed consent from the patient and appropriate permissions from the ethics committee were obtained.

## Discussion

Tooth autotransplantation has been one of the major foci of ongoing research regarding tooth replacement options. One of the main concerns about any tooth replacement option is the prognosis and the longevity of the replacement. Kafourou et al. [7] retrospectively investigated the cases of TAT in their clinic that were performed on patients of up to 16 years of age and reported an 87.6% success rate and a 94.4% survival rate. Of the teeth eligible for pulpal revascularization, 75.6% showed continued pulpal vitality and root formation. The mean follow-up period was 2.6 years, with some cases even reaching almost 10 years of follow-up. They concluded that TAT on children and adolescents has an excellent success rate and is a suitable choice whenever indicated [7]. Barcellos et al. [14] assessed the outcome of 43 TATs with a mean of 9.6 years of follow-up. To be considered as successful, the tooth had to be asymptomatic, free of periapical radiolucency and signs of root resorption, have a healthy periodontium, and have acceptable esthetics. Based on these criteria, 79% of cases were considered to be successful, and the overall survival rate was 97.7% (only 1 tooth loss). The most frequently found abnormalities were periapical radiolucency and external root resorption, all of which seemed to be stable and not indicative of a need for extraction. A meta-analysis done by Machado et al. [15] found a long-term overall survival rate of 81% for TAT, with follow-up periods ranging from 6 to 41 years. They also estimated a 4% prevalence for external root resorption and ankylosis and concluded that the survival rate is excellent considering the follow-up period [15].

Several factors are associated with a more favorable outcome and overall success of TAT [4]: patient’s age, sex, and developmental stage; alveolar support and dimensions of bone at the recipient site; surgical technique; root development of the donor tooth; handling of the donor tooth; the method of donor tooth stabilization; and post-op care. Although immature teeth with an open apex have a greater chance of success with the ideal root being half to full root length with a 1mm open apex, tooth autotransplantation with complete root formation is a favorable treatment with rare failure, root resorption, and ankylosis [12][16]. Many studies indicate that the most important prognostic factor is probably the preservation of periodontal ligament cells’ viability [17][18][19], as this factor directly dictates the periodontal healing of the transplanted tooth. Periodontal healing is the ultimate factor that dictates the overall long-term prognosis of the transplanted tooth, since it is directly correlated with external root resorption and ankylosis, which are irreversible most of the time [15], whereas most of the undesirable outcomes of pulp healing can be managed by RCT [6][7]. Minimal extraoral time, keeping the PDL hydrated, and the removal of the pulp help reduce the probability of infection, inflammation, and the resultant root resorption. In this case, the donor tooth had to undergo RCT since root apices were closed and a high risk of pulp necrosis and subsequent root resorption was present.

To minimize the damage to PDL, the extraction of the donor tooth should be performed as atraumatically as possible, and the time period in which the donor tooth is kept out of the alveolus has to be kept as low as possible. If any additional extraoral procedures, such as root canal treatment, have to be performed on the donor tooth, the tooth has to be either constantly irrigated by a physiologic solution or the root portion has to be immersed in a physiologic solution [18]. To meet these criteria, teeth with severe root curvatures, abnormal root morphologies, and complex root canal systems that preclude atraumatic extraction or hinder timely and quick root canal treatment are not proper candidates to be TAT donors [18][20].

In the present case, the radiographic examination revealed a conical root morphology (suitable for atraumatic extraction) with minimal root canal system complexity (suitable for a quick and straightforward root canal treatment). This third molar had no chance of complete eruption into functional occlusal height due to the lack of adequate arch space and the mesial angulation, making it a suitable choice for autotransplantation. After the extraction, the donor tooth was held from the crown by a forceps suitable for minimizing the damage to the periodontal ligament (a Young Tongue Forceps). Previous studies either held the tooth with a regular extraction forceps or sterile gauze, both of which run the risk of further damage to PDL cells. The tongue forceps used in this study minimized the risk of PDL damage as it prevents crown trauma and slippage owing to the elasticity and ribbing of the rubber part, in addition to providing a locking mechanism to ease the handling of forceps. The Young forceps has been used in general medical procedures, botany, and animal studies. Nevertheless, for the first time, we used it in dental procedures and tooth handling [21][22][23].

Because of completed root formation, the donor tooth should be root canal treated extraorally or intraorally after transplantation. Due to instability and mobility and possible damage to healing gingiva during RCT and rubber dam placement, extraoral RCT was done. In closed-apex donor teeth, immediate intraoperative or ex vivo RCT is recommended (the success rate was less than 28% without RCT). Meta-analytical evidence indicates that postoperative in vivo RCT on third molars frequently fails to achieve hermetic obturation due to complex root canal anatomy, often leading to persistent pulpal necrosis and subsequent periapical inflammation. It is often emphasized that ex vivo RCT must maintain PDL cell viability through strict moisture preservation, aseptic protocols, and atraumatic handling. If these conditions cannot be guaranteed, RCT should be deferred to 3-6 months post-transplantation when initial osseointegration stabilizes the tooth against micro-vibrations from canal instrumentation [20][24].

. The tooth was continuously irrigated with HBSS while the RCT procedure was being carried out, accompanied by intermittent immersion of roots in HBSS whenever irrigation should not have been performed. The RCT procedure and the amalgam filling of the access cavity took approximately 17 minutes to finish, increasing the probability of preserving PDL cell viability and the chances of transplantation success. While generally extraoral procedures in TAT are less favored, due to a more difficult access for the second molar compared to the more anterior teeth, the possibility of a tooth clamp damaging the still healing periodontium, and the anticipation of a straightforward and quick extraoral treatment, the RCT was carried out extraorally before transplantation.

The success of TAT is usually evaluated based on multiple criteria, some of which are [6][7][18]: absence of pain and discomfort; absence of periodontal inflammation and pathology such as pockets; absence of radiographic signs of external root resorption; presence of distinctive lamina dura and PDL space; normal mobility; continuation of root development; and acceptable esthetic outcomes. Even though the transplanted tooth can still function while not meeting some of the aforementioned criteria, it will be counted as tooth survival and not a success. At the 12-month follow-up for the present case, the transplanted tooth met all criteria for success, although it can be argued that a 12-month follow-up period is relatively short, and further monitoring of the transplanted tooth is required to assess its long-term prognosis. This case report is among the first studies to utilize a cold ceramic sealer in TAT. Additional studies that include more samples and longer follow-up periods are required to assess the long-term outcome of TAT, especially those utilizing cold ceramic sealers and extra-oral procedures carried out while holding the tooth using the Young Tongue Holding Forceps.

## Conflict of Interest

None.
